# Symptoms and Clinical Course of EHEC O104 Infection in Hospitalized Patients: A Prospective Single Center Study

**DOI:** 10.1371/journal.pone.0055278

**Published:** 2013-02-27

**Authors:** Sebastian Ullrich, Phillip Bremer, Christine Neumann-Grutzeck, Helge Otto, Christoph Rüther, Cay Uwe von Seydewitz, Gerd Peter Meyer, Keihan Ahmadi-Simab, Joachim Röther, Barbara Hogan, Wolfgang Schwenk, Roman Fischbach, Jörg Caselitz, Jochen Puttfarcken, Susanne Huggett, Petra Tiedeken, Jordan Pober, Nancy C. Kirkiles-Smith, Friedrich Hagenmüller

**Affiliations:** 1 Department of Medicine I (Gastroenterology), Asklepios Klinik Altona, Hamburg, Germany; 2 Department of Medicine II (Oncology and Haematology), Asklepios Klinik Altona, Hamburg, Germany; 3 Department of Medicine III (Cardiology, Angiology and Pulmonology/Intensive Care), Asklepios Klinik Altona, Hamburg, Germany; 4 Department of Medicine IV (Rheumatology, Immunology and Nephrology), Asklepios Klinik Altona, Hamburg, Germany; 5 Department of Neurology, Asklepios Klinik Altona, Hamburg, Germany; 6 Emergency Department, Asklepios Klinik Altona, Hamburg, Germany; 7 Department of Surgery I (General and Visceral Surgery), Asklepios Klinik Altona, Hamburg, Germany; 8 Department of Radiology, Neuroradiology and Nuclear Medicine, Asklepios Klinik Altona, Hamburg, Germany; 9 Department of Pathology, Asklepios Klinik Altona, Hamburg, Germany; 10 MEDILYS, Laborgesellschaft mbh, Hamburg, Germany; 11 Dialysezentrum Hamburg-West, Hamburg, Germany; 12 Department of Immunobiology, Pathology, and Dermatology, Yale University School of Medicine, New Haven, Connecticut, United States of America; 13 Department of Immunobiology, Yale University School of Medicine, New Haven, Connecticut, United States of America; Mario Negri Institute for Pharmacological Research and Azienda Ospedaliera Ospedali Riuniti di Bergamo, Italy

## Abstract

**Objectives:**

Shiga-toxin producing O157:H7 Entero Haemorrhagic *E. coli* (STEC/EHEC) is one of the most common causes of Haemolytic Uraemic Syndrome (HUS) related to infectious haemorrhagic colitis. Nearly all recommendations on clinical management of EHEC infections refer to this strain. The 2011 outbreak in Northern Europe was the first to be caused by the serotype O104:H4. This EHEC strain was found to carry genetic features of Entero Aggregative *E. coli* (EAEC) and extended spectrum β lactamase (ESBL). We report symptoms and complications in patients at one of the most affected centres of the 2011 EHEC O104 outbreak in Northern Germany.

**Methods:**

The courses of patients admitted to our hospital due to bloody diarrhoea with suspected EHEC O104 infection were recorded prospectively. These data include the patients’ histories, clinical findings, and complications.

**Results:**

EHEC O104 infection was confirmed in 61 patients (female = 37; mean age: 44±2 years). The frequency of HUS was 59% (36/61) in our cohort. An enteric colonisation with co-pathogens was found in 57%. Thirty-one (51%) patients were treated with plasma-separation/plasmapheresis, 16 (26%) with haemodialysis, and 7 (11%) with Eculizumab. Patients receiving antibiotic treatment (n = 37; 61%) experienced no apparent change in their clinical course. Twenty-six (43%) patients suffered from neurological symptoms. One 83-year-old patient died due to comorbidities after HUS was successfully treated.

**Conclusions:**

EHEC O104:H4 infections differ markedly from earlier reports on O157:H7 induced enterocolitis in regard to epidemiology, symptomatology, and frequency of complications. We recommend a standard of practice for clinical monitoring and support the renaming of EHEC O104:H4 syndrome as “EAHEC disease”.

## Introduction

In May and July 2011 Germany experienced an Entero Haemolytic *Escherichia coli* (EHEC) O104 infection outbreak. The Robert Koch Institut (RKI), a Federal Institute within the portfolio of the Federal Ministry of Health, reported 2987 cases of Shiga-toxin mediated gastroenteritis [1]. The outbreak was declared to have been terminated on July 26^th^ 2011. Most cases occurred in Northern Germany, and fenugreek seeds from Egypt were suspected (but not established) as the source of infection [1], [2]. Recent reports have focused on bacteriology, epidemiology, and the incidence of the Haemolytic Uraemic Syndrome (HUS), a major complication of EHEC gastroenteritis [3]–[5]. Most former EHEC outbreaks were related to the O157:H7 strain of *E. coli*, while the recent cases have been caused by the O104:H4 strain. Prior to the recent episode in Germany, only minor outbreaks related to EHEC O104 H4 had been reported [6], [7]. The current O104:H4 strain is characterised by the expression of Shiga-toxin 2 and “Extended Spectrum β-Lactamase“ (ESBL) [8]. Genome sequence analysis has demonstrated that the current pathogen represents a new phenotype which combines the characteristics of EHEC and Entero Aggregative *Escherichia coli* (EAEC) [9]. This finding may explain the strong adherence of the O104:H4 strain to the colon mucosa and the enhancement of virulence. Brzuszkiewicz et al. suggest the term Entero Aggregative Haemorrhagic *Escherichia coli* (EAHEC) to describe this new phenotype [3].

HUS, first described in 1955 [10], is characterized by the triad of acute renal failure, haemolytic anaemia, and thrombocytopenia, mostly affecting children [11]. The recent EHEC episode has been complicated by HUS in 25% of patients [4]. However, in contrast to previous outbreaks, young female adults have been affected in the majority of cases, whereas pediatric cases remained rare [4]. Earlier O157:H7 outbreaks found HUS in only 7% of adult patients [12].

The evidence base for the treatment of EHEC enterocolitis and HUS is limited [13]. Recommendations rely on retrospective analyses of outbreaks (mostly O157:H7) and conclusions from *in vitro* research. The use of antibiotics is controversial [14]–[18]; some *in vitro* data suggest that antibiotics could aggravate the course of the disease by enhancing Shiga-toxin production [11]. Plasmapheresis for the treatment of HUS is used on an empiric basis and is recommended by the German Society of Nephrology in case of neurological complications and/or rapid onset of HUS accompanied by thrombocytopenia [19]. The successful use of Eculizumab, a monoclonal antibody that prevents the activation of the fifth component of the complement cascade, for the treatment of HUS in children has been recently reported during the current EHEC outbreak [20]. Here we describe the clinical presentation and course of patients with infection by EHEC O104:H4 who were hospitalized in our institution in May to July 2011.

## Materials and Methods

### Patients

On the 14^th^ of May 2011 two patients with bloody diarrhoea were admitted to our hospital. These two patients were among the first cases of the recent EHEC outbreak reported to the RKI. During the following 41 days, a total of 61 patients with bloody and/or painful diarrhoea due to EHEC colitis were hospitalized at our institution.

On May 19^th^ the RKI released the first information on an EHEC infection outbreak in Germany. From this date onward, we prospectively documented standardized parameters of symptoms, clinical course, and complications of all our hospitalized patients until their discharge. Inclusion criteria were diarrhoea (≥3 stools/24 h) at time of admission, positive stool testing for EHEC and/or signs of HUS. Data on the patients` history, previous medication, general and abdominal symptoms, physical findings, frequency and quality of stools, blood chemistry, ultrasonic, and radiologic findings were collected at admission, discharge, and at defined time points (onset of HUS, initiation of antibiotic treatment and plasma-separation). From 14^th^ of May until July the 26^th^ laboratory data of all in-patients were recorded at least every second day, in case of HUS daily. All patients gave their written consent in this study; the study protocol was approved by the ethical committee of the Chamber of Physicians Hannover (No.: 1123–2011).

### Definition of Complications

Symptoms and findings other than bloody diarrhoea and abdominal pain that compromised the general condition of patients or resulted in medical intervention and/or prolongation of hospitalisation were recorded as complications. HUS was defined as rise of serum creatinine above >0.5 mg/dl, thrombocytopenia <150/nl, signs of haemolysis with anaemia, and schistocytes [21].

### Microbiologic Testing

Stool cultures for EHEC were performed at least three times. *E. coli* colonies were tested for their ESBL status. All cultures which showed growth of *E. coli* underwent a screening for the presence of Shiga-toxin 1, Shiga-toxin 2, and Intimin-gene using a PCR-technique. The PCR were performed from grown *E. coli* culture on selective agar plates (BRILLIANCE UTI, Oxoid). If possible 20 colonies at minimum were picked and transfered into 500 µL sterile water (PCR grade), incubated for 10 minutes at 95°C and centrifuged for 5 minutes at maximum speed (>12000×g) in a microliter centrifuge. 5 µL of the supernatant were pipetted into the PCR master mix.

The PCR and subsequent DNA-hybridisations were performed in accordance with the manufacturer’s instructions (GenoType EHEC, Hain Lifescience GmbH, Nehren, Germany).

The test system detects the toxin genes Shiga-toxin 1 and 2 (EHEC) and the Intimin-gene (enteropathogenic *E. coli*).

In addition, all stool samples were tested for other enteropathogenic bacteria and viruses, such as other pathogenic *E. coli*, *Clostridium*, *Salmonella*, *Shigella*, *Campylobacter jejuni,* and Noro−/Adeno-virus. Patients suffering from bloody diarrhoea and HUS who had three negative stool cultures for EHEC and Shiga-toxin were considered as false negative stool cultures.

### Fluid Management and Analgesic Therapy

All patients received an extensive intravenous substitution of fluids, up to five litres a day, depending on renal and cardiac function [22]. Metamizol, Paracetamol or Piritramid were used for analgesia; opiod-analgesics were not used to avoid inhibition of peristalsis. The majority of patients received peroral gut lavage with 1 l/d PEG-based solutions, to accelerate elimination of Shiga-toxin from the bowel.

### Antibiotic Treatment

Recommendations for the use of antibiotics in EHEC infection changed during the course of the outbreak. Initially, a potential negative influence on the course of the disease was presumed based upon uncontrolled data [16]–[18], [23]–[26]. During the ongoing outbreak the German Society of Infectiology [27] pleaded for a more liberal use of antibiotics. Recommendations were altered and patients were additionally treated with daily oral administration of Rifaximin, as earlier reports demonstrated that this agent does not increase Shiga-toxin 1/2 production *in vitro* [24]. Patients were either treated at time of admission or for persisting EHEC colonization. In case of bacteria- associated complications, additional antibiotic treatment was initiated according to the clinical findings and the bacteriologic results.

### Plasma-separation/Plasmapheresis and Dialysis

Plasma-separation (Spectra Optia®), or plasmapheresis (Microplas® plasmafilter) was initiated in cases of HUS, isolated rapid onset of thrombocytopenia, renal failure, haemolysis or severe neurological symptoms. Plasma-separation was performed once a day. The quantity of fresh frozen plasma used was adapted to patients’ body weight: <70 kg: 10 units; >70 kg: 12 units. Systemic steroid treatment was only applied in case of allergic reactions. Procedures were stopped when laboratory signs of HUS and neurological status recovered. Dialysis was used in cases of progressive renal failure and was stopped individually with return of renal function.

### Eculizumab

The preliminary report of the successful use of Eculizumab in 3 children with Shiga-toxin associated HUS was published On May 25, 2011 [20]. Thereafter patients with severe neurological complications refractory to standard plasma-separation were treated with Eculizumab. Initially patients received a single dose of Eculizumab [600 mg] following administration of Rifampicin for meningitis prophylaxis. Patients were re-evaluated after 36 hours. In case of treatment failure, daily plasma-separation with subsequent administration of Eculizumab [300 mg] was administered for three more days. If repeated re-evaluation showed no clinical benefit, treatment was switched to plasma-separation twice daily.

### Histology and Immunohistochemistry

Colonic biopsies were obtained from three patients and analyzed by standard hematoxylin and eosin staining of paraffin-embedded sections. Samples from one patient were further analyzed by immunohistochemistry of paraffin-embedded sections, performed as follows: Sections were cut and baked onto slides at 60°C for 4 hours. Slides were deparaffinized with two sequential immersions into 100% xylene for 5 minutes followed by rehydration in graded alcohols (100%, 95% and 80%) for 5 minutes each. After deparaffinization and rehydration the slides were put into dH2O. Sections were next subjected to antigen retrieval by heating in citrate buffer (10 mM Citric acid, 0.05% Tween, pH 6.0) for 30 minutes at 95 to 100°C. Slides were allowed to cool and then rinsed with PBS/0.05% Tween for 5 minutes. Sections were blocked for 30 minutes in normal serum blocking solution (Bloxall Blocking Solution, Vector Labs Burlingame CA) and then incubated with primary antibodies overnight at 4°C. Antibodies used were mouse anti-human CD31 (Clone JC70A, DAKO Carpinteria CA) and mouse anti-human VCAM-1 (Clone 6G9, Novus Biologicals Littleton, CO) and IgG1k isotype control (BD Pharmingen, San Diego CA). The sections were rinsed twice in PBS/0.05% Tween and then incubated with a biotin-conjugated goat anti-mouse IgG secondary for 60 minutes (Jackson Immunoresearch, West Grove PA) followed by sequential rinses and incubation with avidin and biotinylated HRP (Elite ABC Vectastain Kit, Vector, Burlingame, CA), all at room temperature. The peroxidase label was developed using 3-aminoethylcarbazole (Red AEC kit, Vector) and the sections were counterstained with hematoxylin (Sigma, St. Louis MO).

### Statistical Analysis

Data are presented as means±SEM or median and ranges, using SPSS 12.0 for windows (SPSS Inc. Chicago, USA).

## Results

### Patient Characteristics and Microbiologic Findings

Sixty-one patients were enrolled in this study (Table 1) based upon hospital admission for bloody (n = 57; 93%) or severe (n = 4; 7%) diarrhoea. Twelve patients (20%) fulfilled the criteria of HUS at time of admission. The mean age of patients was 44±2 years, 37 females and 24 males. The mean interval between onset of diarrhoea and admittance was 3±0.4 days, the mean duration of hospitalisation 23±2 days. All patients were treated in a regular clinical setting; temporary intensive care was needed in 22 cases (36%). 59 (97%) patients had a positive test for Shiga-toxin-2. 19 (31%) patients tested positive for the Intimin-gene. The stools of two patients with bloody diarrhoea and HUS were tested negative for EHEC. All tested EHEC cultures belonged to the O104:H4 strain and an extended-spectrum beta-lactamase (ESBL) type CTX-M-15 with the upstream insertion sequence ISEcp1 and a beta-lactamase type TEM-1 was detected in all isolates. The resistance genes blaCTX-M-15 and blaTEM-1 were localized on a transferable plasmid (IncI1 replicon, about 90 kbp). 35 (57%) patients had co-infections with either *Clostridium difficile* or Noro-virus (26 patients each), 18 (30%) patients tested positive for both pathogens. Another 2 patients suffered from co-infection with either *Campylobacter jejuni* (n = 1), or Adeno-virus (n = 1). Patient with co-infections did not suffer from specific symptoms, therefore first co-infections were diagnosed by accident, and screening for co-infections was continuated during outbreak. All co-infections were diagnosed within 3 days after admission so that nosocomial infection seems unlikely.

**Table 1:** Patients characteristics, preexisting disease, symptoms on admission, stool microbiology.

Patients	n = 61 (%)
**Mean age [years±SEM]**	44±2
**Men/Women**	24/37 (39/61)
**Diarrhoea**	61 (100)
**Bloody diarrhoea**	57 (93)
**Abdominal pain**	54 (89)
**Nausea**	34 (56)
**Vomiting**	25 (41)
**Fever**	6 (10)
**HUS at time of admission**	12 (20)
**Shiga-toxin 2 positive**	59 (97)
**Intimin-gen positive**	19 (31)
**Intestinal co-Infection**	35 (57)
** Clostridium difficile**	26 (43)
** Norovirus**	26 (43)
** Campylobacter jejuni**	1 (2)
**Preexisting renal disease**	1 (2)
**Preexisting hypertension**	9 (15)

### Early Symptoms and Findings

**Figure 1:**
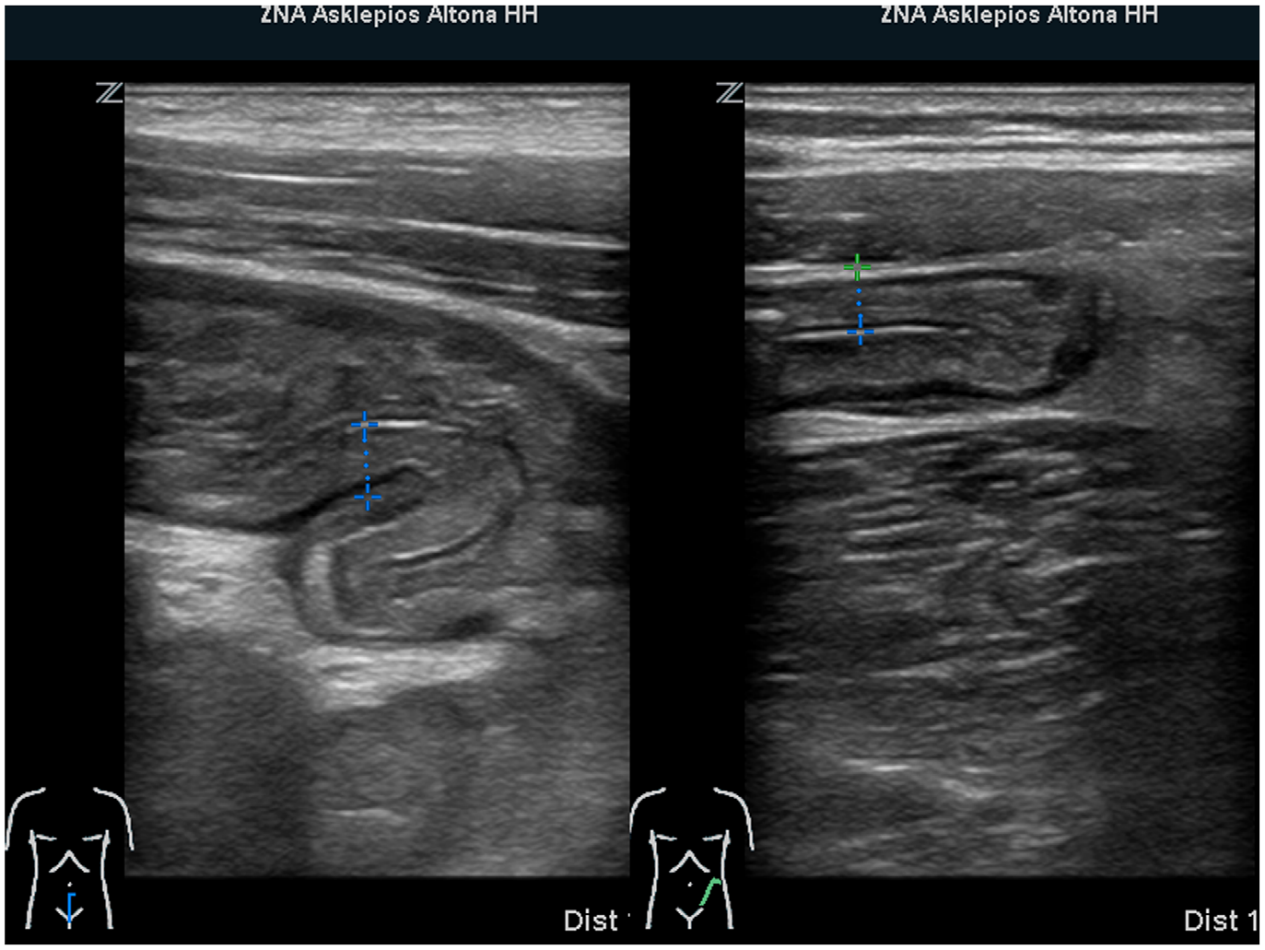
Typical ultrasound image in EHEC O104 infection. left sided colitis with marked thickening of the colonic wall.

**Figure 2:**
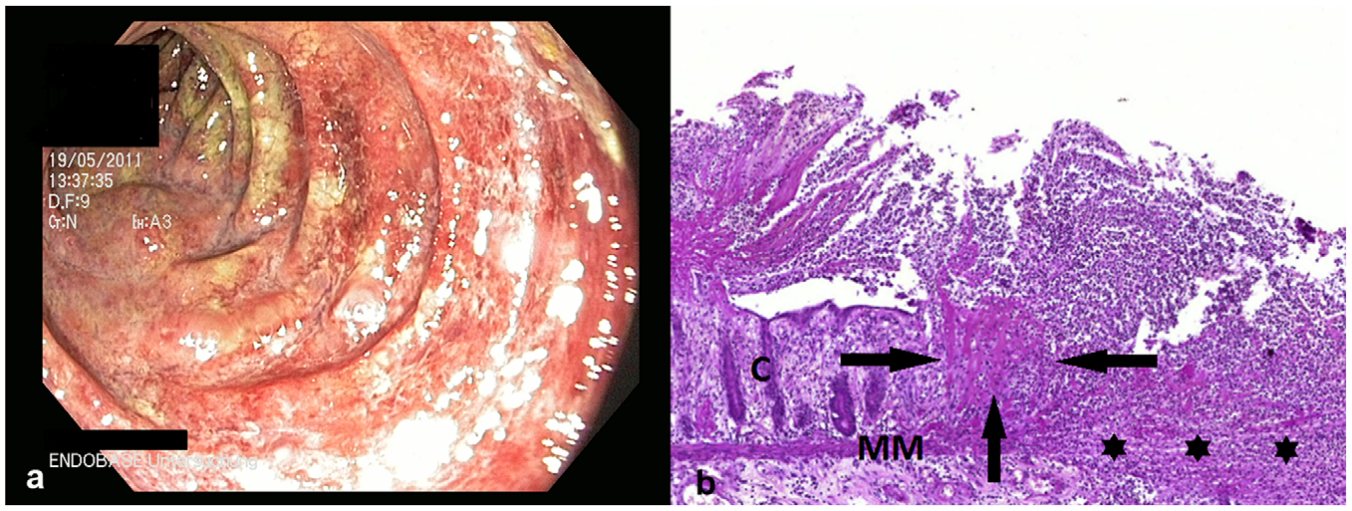
Endoscopic image (a) of EHEC O104 induced hemorrhagic necrotizing colitis and corresponding histology (b). PAS staining of colon mucosa after surgical resection: massive granulocyte infiltrations with colonic crypts (C) and severe ulceration: disruption (asterix) of muscularis mucosae (MM), fibrin deposits (arrows) and edema.

**Figure 3:**
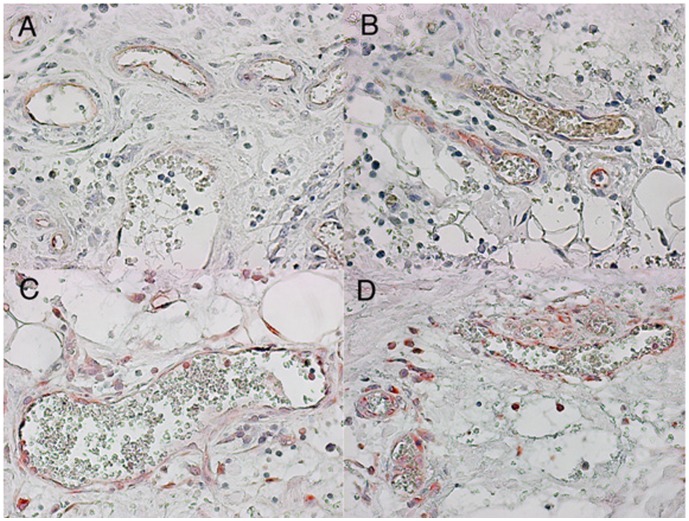
Photomicrographs of two separate gut sections from a patient with EHEC colitis. Panels (A) and (B) are stained with CD31 to enumerate endothelium lining the vessels (40× magnification). (C) and (D) are stained to show VCAM-1 expression in endothelium, indicating inflammatory activation (40× magnification).

The initial symptom in most patients was spasmodic abdominal pain of the lower abdomen, with emphasis of the left side, often accompanied by nausea (n = 34; 56%) and vomiting (n = 25; 41%). Fever (n = 6; 15%) was found in a minority of cases. All patients experienced the onset of diarrhoea within a few hours to three days after onset of initial symptoms. The frequency of bloody diarrhoea was 93%. The condition at time of admission commonly included dehydration with distinct fatigue, a general feeling of illness, weakness, and headache. Abdominal findings included a diffuse tenderness of the abdomen with meteorism. Laboratory findings on admission showed a mild elevation of the CRP 35.7±7.2 mg/l and leucocytosis (12.3±0.7/nl) (Table 2). The typical abdominal appearance on ultrasound was that of left sided colitis with marked thickening of bowel wall and ascites (Fig. 1). At the beginning of the outbreak three of the first patients underwent colonoscopy for differential diagnosis of bloody diarrhoea. In these cases, endoscopy showed a severe ulcerative colitis with spontaneous bleeding of the highly inflamed mucosa (Fig. 2a). Histologic examination demonstrated severe inflammation with dense infiltrations of lymphocytes and granulocytes, ulcerative disruption of the epithelial lining and fibrin deposits (Fig. 2b). Immunohistochemistry of blood vessels in areas of inflammation revealed an intact endothelial lining with induced endothelial cell expression of VCAM-1 indicative of inflammatory activation (Fig. 3).

**Table 2:** Stool frequency and laboratory data at different courses of disease.

	Hospital-admissionn = 61	Onset of HUSn = 36	Beginning of plasma-separation n = 33	Discharge n = 60
**Stool frequency [/d]**	21±3	8±2	5±1	1±0
**Hb [g/dl]**	13.7±0.3	12.1±0.3	11.4±0.3	10.6±0.2
**Thrombocytes [/nl]**	218±12	78±6	76±14	313±16
**CRP [mg/l]**	35.7±7.2	71.4±10.5	77.9±12.5	10.4±2.1
**Creatinine [mg/dl]**	1.3±0.1	1.7±0.2	1.9±0.2	1.2±0.1
**LDH [U/l]**	403±72	793±84	1033±106	226±12

(Mean±SEM); reference levels: leucocytes: 3.6–10/nl, Hb: 13–15 g/dl, thrombocytes: 150–450/nl, CRP: <5 mg/l, creatinine: 0.5–1.0 mg/dl, LDH: <250 U/l.

### Further Course, Complications, and Therapy

**Figure 4:**
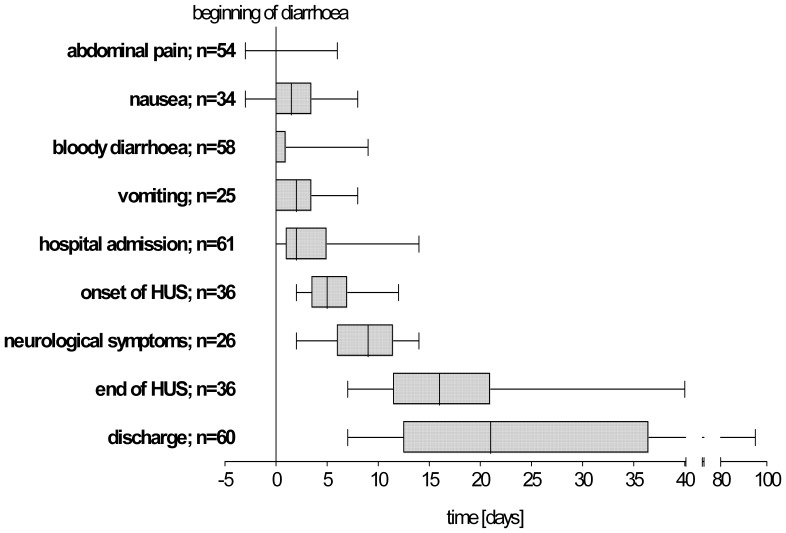
Time course of symptoms, complications, admission, and discharge in relation to the beginning of diarrhoea. [range, 25^th^–75^th^ -percentile, median].

Seventeen patients (28%) with diarrhoea improved continuously and could be discharged free of symptoms after 7±1 days. The remaining 44 (72%) patients developed complications. In many cases complications were preceded by a stagnation of bowel movements. The time-wise sequence of symptoms and complications is shown in Fig. 4. The longest interval between onset of diarrhoea and onset of complications was 14 days.

**Figure 5:**
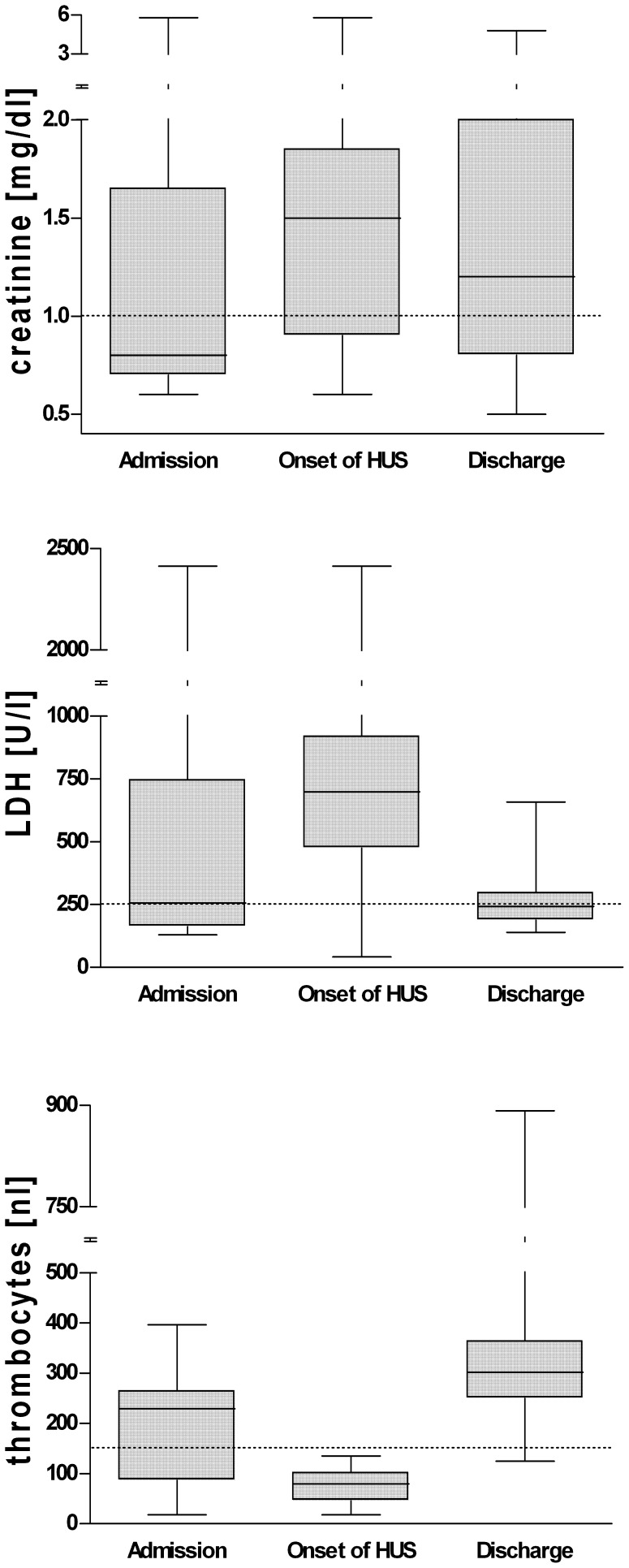
Development of serum creatinine, LDH, and thrombocytes in 36 patient suffering from HUS. [range, 25^th^–75^th^ percentiles, median, reference levels].

The most frequent and severe complication was HUS which developed in 36 cases (59%; male/female: 11/25). In 17 (47%) out of 36 HUS-patients diarrhoea had already ceased at time of the onset of HUS. All patients with HUS suffered from typical haemolysis, progressive renal failure, and thrombocytopenia. The cumulative laboratory findings of HUS patients are shown in Fig. 5. The mean duration of HUS was 12±1 days. 33/36 (92%) patients with HUS were treated with plasma-separation (median: 10 cycles (3–50), median duration: 9 days (2–35)) and dialysis in cases of renal failure (16 patients; 44%). While 17 (47%) patients reached normal levels of the serum creatinine subsequent to HUS, 19 patients displayed prolonged kidney damage, indicated by sustained elevations of serum creatinine (>1.2 mg/dl) and/or reduced glomerular filtration rate (GFR). Two patients had to continue dialysis at time of discharge. All HUS patients developed a severe capillary leak syndrome with a rapid onset along with first laboratory signs of HUS and had therefore to be treated with extensive replacement of fluids. Besides generalized oedema, most patients suffered from pleural effusions (29/36; 81%) and ascites (28/36; 78%).

Neurologic complications (n = 26/61; 43%) occurred 4 days (2–11) after the diagnosis of HUS. Patients presented with epileptic seizures (n = 13; 50%), oculomotor dysfunction (n = 19; 73%), neuropsychiatric syndromes (n = 18; 69%), disorientation (n = 15; 57%), somnolence (n = 11; 42%), aphasia (n = 9; 34%), tremor (n = 9; 34%), cortical blindness (n = 3; 11%), choreatic syndrome (n = 1; 4%). In nearly all cases the initial neurological symptoms progressed within hours towards complex syndromes. While most neurological complications affected patients with HUS (n = 23), some also occurred independently from HUS (3 cases). All patients with seizures received anticonvulsive treatment, which was discontinued within weeks after discharge. Paresis was also observed (n = 7; 27%) in different stages of the disease ranging from transient attacks to severe hemiparesis. After discharge, two patients suffered from persistent neurological damage (cortical blindness, choreatic syndrome).

Seven patients with neurological symptoms did not improve or progressed despite repeated plasma-separation and therefore received Eculizumab. As none of these patients seemed to benefit from this regimen, all patients were switched to plasma-separation twice daily. The number of patients treated was too small for statistical analysis of outcomes.

Overall 37 (61%) patients received antibiotic treatment for coinfections with *Clostridium difficile* or infectious complications separate from EHEC enterocolitis (28× Metronidazol, 11× carbapenemes, 5× cephalosporine, 4× Ciprofloxacin, 4× aminopenicillin, 3× Penicillin, 1× aminopenicillin/betalactamase-inhibitor, 2× Piperacillin/Tazobactam, 1× Nitrofurantoin, 1× Daptomycin, and 1× Vancomycin). No aggravation of the clinical course was observed in any case after administration of antibiotics.

During the later course of the outbreak 5 patients were treated with peroral Rifaximin on admission with the intention to prevent HUS, which occurred in only one of these cases. The number of patients so treated was not large enough to allow statistical analysis. Three patients received Rifaximin in order to eliminate persisting EHEC colonisation, which was not successful in any patient.

PEG-based lavage was tolerated by 51/61 (84%) patients. Judgments regarding the efficacy of this procedure cannot be drawn.

Temporary or prolonged hypertension occurred or was exacerbated in 48% of patients. Most of these patients suffered from HUS. Twenty-one (34%) patients suffered from newly acquired or aggravated arterial hypertension (RR>140/90 mmHg) on discharge.

**Figure 6:**
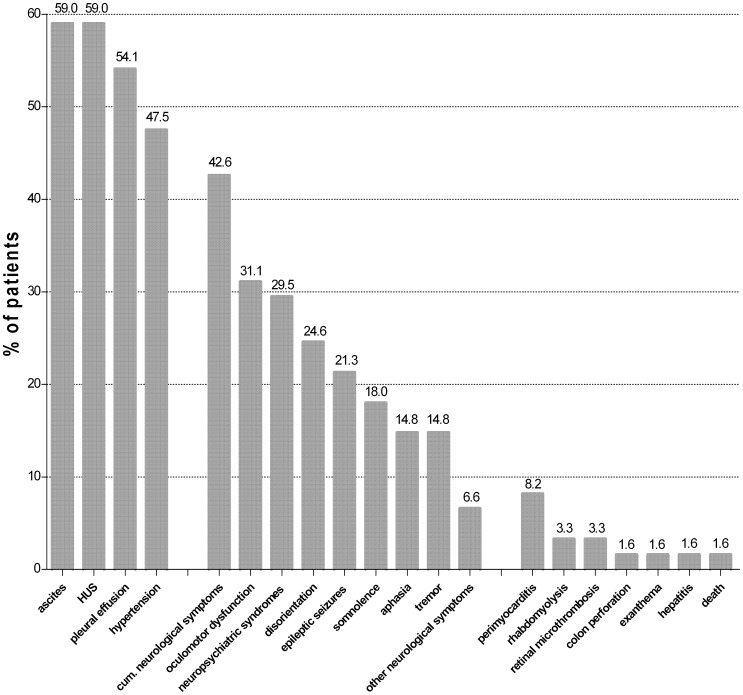
Complications in 61 patients with EHEC O104 infection. cum: cumulative; other neurological symptoms include: cortical blindness (n = 3) and choreatic syndrome (n = 1).

Uncommon complications were hyperlipasaemia (13%), perimyocarditis (8%), retinal microthrombosis (3%), rhabdomyolysis (3%), and skin rash (2%) and were in most cases associated with HUS. A listing of all complications is shown in figure 6.

**Figure 7:**
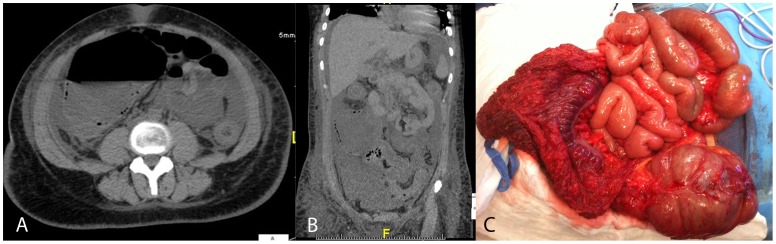
Patient with EHEC O104, Noro-virus, and Clostridium difficile co-infection. CT scan showing massive swelling of the intestinal wall, extreme dilation of the right colon with intramural air (a and b). Intraoperative situs with extreme wall thickening of the ascending colon and incipient perforation of the cecum (c).

One patient with co-infection with Clostridium difficile underwent subtotal colectomy for necrotizing colitis with peritonitis and incipient perforation (see Fig. 7a–c).

A 83-year-old female patient, suffering from chronic obstructive pulmonary disease died 39 days after hospital admission and 26 days despite successful treatment of HUS because of respiratory failure caused by pneumonia associated with pleural effusions after intensive care.

## Discussion

The aim of this study was to characterise symptoms and clinical course of EHEC O104:H4 infection at the time of the recent outbreak in Germany. As only hospitalized patients were analysed, our results do not reflect the full spectrum of the epidemiology. The observed patient characteristics, symptoms, and complications differ from earlier reports EHEC outbreaks, which focused predominantly on EHEC O157:H7 infections [28]–[34]. In accordance with the first epidemiologic analysis [4], [35] our data confirm young adult female patients to be the largest group affected by the 2011 EHEC-induced disease in Germany (61%). Early symptoms are comparable to the onset of EHEC 0157 infections [29]. The most common symptom leading to hospital admission was bloody diarrhoea. HUS represents the most frequent (59%) and severe complication, a conclusion consistent with results of the first epidemiologic analysis [4]. This differs markedly from the reported incidence of 10–15% HUS in EHEC 0157 infections [28]. Severe neurological manifestations occurred in many patients (43%) and coincided with HUS in most cases. Reports of EHEC 0157 infections described neurological complications in 30% of HUS patients [30]–[32], [36], [37] with comparable manifestations, but mainly in children [38]. We also observed severe neurological complications in non-HUS patients (3/26). The sudden onset and the severity of neurological symptoms require intensive observation to ensure adequate treatment.

Complications can develop independently from diarrhoea, as found in 17/36 (47%) HUS patients. Rapid stagnation of bowel movements seemed to indicate the development of complications. In many cases the time gap between cessation of diarrhoea and onset of complications was either misleadingly long (up to 6 days), or complications developed within hours and resulted in the immediate need for intensive care. These observations prompted us to adapt our care in terms of an intensified monitoring at frequent intervals (“Altona EAHEC Monitoring Standard”; Table 3). Search for manifestations of thrombotic microangiopathy should contribute to early detection of complications before they become clinically evident e.g. cardiac arrhythmia. This approach, including an extensive fluid-management, may have helped to keep mortality in our cohort (3% of HUS patients) below rates reported earlier (9%) [33], [34]. In contrast to earlier reports [17], [28] we could not observe any case of deterioration attributable to antibiotic treatment. A recent publication on the use of Azithromycin in EHEC O104:H4 infection found no increase in frequency of HUS or worsening of EHEC related symptoms. Treatment with Azithromycin was correlated with a shorter time of EHEC colonisation [15]. In vitro data indicate different effects on Shiga-toxin production depending on the antibiotic agent used: Ciprofloxacin induces Shiga-toxin production while Meropenem, Azithromycin, Tigecyline, and Rifaximin do not influence Shiga-toxin production [39].

**Table 3:** The “Altona EAHEC Monitoring Standard.”

	Diagnostics
**At hospital admission**	Stool culture including other pathogenic E. coli, Clostridium, Salmonella, Shigella, Campylobacter, Noro−/Adeno-viruses
	Abdominal and pleural ultrasound
	ECG
	Blood chemistry*, including: troponine, albumin
	Stool frequency
**In case of stagnating bowel movements**	Twice daily blood chemistry*, once a day: full blood count, LDH, creatinine
	Daily ultrasound examination
	Monitoring of diuresis
	Daily monitoring of bodyweight
**In case of HUS**	Twice daily blood chemistry*, once a day: full blood count, LDH, creatinine
	Monitoring of diuresis
	Daily ECG
	Twice daily neurological examination
	Differentiation of proteinuria
	Blood pressure
**Subsequent to HUS**	Blood chemistry* according to the clinical course
	Blood pressure
	Quantification of proteinuria
	Stool culture
	Ultrasound examination
**Specific symptoms/suggestive test result**	Echocardiography
	Ophthalmologic examination
	Dermatologic examination
	EEG
	Chest-X-Ray
	Cranial-MRI
	Abdominal-X-Ray/Computer tomography
	ECG-Monitoring

Proposal of a diagnostic standard of management in patients with EHEC O104:H4 infection;

* CRP, electrolytes, creatinine, urea, lactate dehydrogenase (LDH), haptoglobin, transaminases, lipase, creatinine kinase, full blood count including fragmentocytes, partial thromboplastin time (PTT), international normalized ratio (INR).

Because of the limited number of patients, statistical analysis of the effectiveness of therapeutic procedures as plasma-separation, treatment with Eculizumab, and antibiotic treatment with Rifaximin are not appropriate. Further analyses of larger numbers of patients will soon be available to clarify these questions.

A surprising finding in our cohort was the unexpectedly high number of coincident infections of EHEC with *Clostridium difficile* and Noro viruses. Overall we found no correlation between coincident infections, the clinical course and EHEC related complications. It has to be noted that the patient that underwent colonic resection due to toxic megacolon was positive for *Clostridium difficile* and abdominal CT revealed gas inclusions in the colonic wall. One case of a toxic megacolon related to a single infection with EHEC 0157 has been described earlier [40]. Patients with coincident Noro virus infections seldom suffered from vomiting. Interestingly, earlier reports describe the detection of *Clostridium difficile*, Rota- and Norovirus in patients suffering from EHEC enterocolitis [41]–[44]. The high prevalence of co-infections in our cohort may simply reflect the high number of tests for different pathogens we repeatedly performed in this prospective study. Further investigations on this topic are needed to understand the relevance of this finding for the course of EHEC O104:H4 infection. We support the recent recommendation to rename the clinical syndrome of EHEC O104:H4 infection as “EAHEC disease” [3] because of its specific clinical course differing markedly from other EHEC infections.

### Conclusion

In contrast to earlier reports on EHEC infections, the recent EHEC O104:H4 outbreak affected mainly young adult females and resulted in a higher number of HUS and neurological complications. EHEC related enterocolitis was associated with an unduly high incidence of enteric co-infections. The diversity of symptoms and organ manifestations, the misleading time gap between cessation of abdominal symptoms and onset of complications as the rapidly changing symptomatology has resulted in our suggestion for an intensified monitoring (“Altona EAHEC Monitoring Standard”). The clinical course of our patients does not confirm earlier concerns about a potentially negative impact of antibiotic treatment. Further analyses are needed to evaluate treatment protocols. The correlation between the genetic and clinical specificity of the EHEC O104:H4 syndrome supports the suggested naming “EAHEC disease”.
